# Riding high: seroprevalence of SARS-CoV-2 after 4 pandemic waves in Manitoba, Canada, April 2020–February 2022

**DOI:** 10.1186/s12889-023-17239-6

**Published:** 2023-12-05

**Authors:** Scotty Duong, Julian Burtniak, Ainsley Gretchen, Anh Mai, Penny Klassen, Yichun Wei, Carla Loeppky, Souradet Y. Shaw, Jared Bullard, Paul Van Caeseele, Derek Riley Stein

**Affiliations:** 1 University of Manitoba, Winnipeg, Canada; 2 Cadham Provincial Laboratory, Manitoba Health, Winnipeg, Canada; 3 Epidemiology & Surveillance, Manitoba Health, Seniors and Active Living, Winnipeg, Canada

**Keywords:** Seroprevalence, SARS-CoV-2, Vaccination, Serology

## Abstract

**Background:**

Canada is emerging from the largest SARS-CoV-2 Omicron wave to date, with over 3.3 million confirmed cases. Unfortunately, PCR confirmed cases illuminate only a small portion of infections in the community and underestimate true disease burden. Population based seroprevalence studies, which measure antibody levels against a virus can more accurately estimate infection rates in the community and identify geographical and epidemiological trends to inform public health responses.

**Methods:**

The Manitoba COVID-19 Seroprevalence (MCS) study is a population-based cross-sectional study to assess the prevalence of SARS-CoV-2 antibodies across the province. Residual convenience specimens (*n* = 14,901) were tested for anti-SARS-CoV-2 nucleocapsid and spike IgG antibodies from April 1, 2020 to February 31, 2022. We estimated the monthly and cumulative prevalence using an exponential decay model, accounting for population demographics, sensitivity/specificity, and antibody waning. This approach generated estimates of natural infection as well as total antibody including vaccine-induced immunity within the community.

**Findings:**

After four waves of the pandemic, 60.1% (95%CI-56.6–63.7) of Manitobans have generated SARS-CoV-2 antibodies due to natural exposure independent of vaccination. Geographical analysis indicates a large portion of provincial prevalence stems from increased transmission in the Northern (92.3%) and Southern (71.8%) regional health authorities. Despite the high mortality rates reported by Manitoba, infection fatality ratios (IFR) peaked at 0.67% and declined to 0.20% following the Omicron wave, indicating parity with other national and international jurisdictions. Manitoba has achieved 93.4% (95%CI- 91.5–95.1) total antibody when including vaccination.

**Interpretation:**

Our data shows that more than 3 in 5 Manitobans have been infected by SARS-CoV-2 after four waves of the pandemic. This study also identifies key geographical and age specific prevalence rates that have contributed greatly to the overall severity of the pandemic in Manitoba and will inform jurisdictions considering reduction of public health measures.

**Supplementary Information:**

The online version contains supplementary material available at 10.1186/s12889-023-17239-6.

## Research in context

Evidence before this study – At the time of submission a search of peer-reviewed publications from 2019–2022 on PubMed with SARS-CoV-2 or COVID-19 and Seroprevalence resulted in 2213 studies. Many of these studies are derived from blood donors 18 + or focus on a specific subgroup (adult, pediatric, or health care workers). We found no studies to date that include all age groups, account for population sampling bias, sensitivity and specificity of the serological assays used, waning immunity, and vaccination. Many studies attempt to address some of these challenges; however, all these factors must be included in order to obtain an accurate estimate of the true natural infection rate in a community.

Added value of this study—The Manitoba COVID Seroprevalence (MCS) study is a population-level seroprevalence study that has overcome several key challenges with interpreting seroprevalence estimates. Accounting for all age groups, vaccination status, and waning antibody have allowed for a more accurate estimate of the true infection burden in the province of Manitoba. Children aged 1–9 years old had the highest prevalence in the province while the elderly had the lowest (60 +). In addition, adjusting for high infection prevalence, resulted in an infection fatality ratio similar to the national average, despite initial indications that Manitoba had one of the highest fatality rates in the country.

Implications of all the available data—The MCS study has revealed a remarkably high rate of natural infection in the community (60.1%). Our data shows that more than 3 in 5 Manitobans have been infected by SARS-CoV-2 after four waves of the pandemic. This study identifies key disparities with respect to geographical and age specific prevalence rates. Despite stringent public health measures, areas within the city of Winnipeg associated with insufficient housing, and poverty, had the highest disease burden. In addition, early vaccine prioritization may have significantly attributed to a low infection fatality rate in the Northern Health Region despite significant transmission. Careful interpretation of seroprevalence estimates with both community demographics and epidemiology are key to gaining accurate insight into how pandemic policies affected the unfolding pandemic.

## Introduction

As of June 2022, there are over 533 million confirmed polymerase chain reaction (PCR) cases of SARS-CoV-2 infection and almost 6.4 million deaths from the virus reported globally. Canada has reported 3.9 million cases and over 41 thousand deaths as of June 2022 [1]. Given the efficient spread of SARS-CoV-2 in the community setting, understanding the true cumulative incidence of the virus is important. By defining the proportion of the population with humoral immunity and the immunologically naive, public health responses to the pandemic can be optimized to address changing trends over time. Determination of anti-SARS-CoV-2 seroprevalence through serological surveillance is central in achieving these major public health goals.

Manitoba identified its first PCR-positive confirmed case of SARS-CoV-2 infection on March 13, 2020, subsequently experiencing four major waves, respectively dominated by the Wild type (March 2020), Alpha/Beta (January 2021), and Delta variants (July 2021), culminating with an Omicron variant bimodal wave (December 2021). In many Canadian provinces the Omicron wave overwhelmed conventional testing capacity [2]. Adoption of rapid antigen testing during the Omicron wave also made obtaining accurate estimates of incidence even more difficult as these tests were not monitored by public health. In addition, changing patterns in testing and public health policy made estimates of the true infection rate extremely challenging. The beginning of the pandemic included travel restrictions within Manitoba, particularly to Northern communities, as well as school closures, limitations on group gatherings, and public mask mandates. In addition, SARS-CoV-2 testing criteria fluctuated from asymptomatic testing to symptomatic testing during all waves of the pandemic. These restrictions gradually relaxed following mass vaccination leading into the Omicron wave. While symptomatic cases are more likely to be identified, asymptomatic to mildly symptomatic cases may go untested (non-healthcare-seeking), and thus are not captured or reported as PCR-confirmed cases [3, 4]. As a result, reported case data largely underestimates the true prevalence of disease in the broader population [3, 5, 6]. Meta-analyses estimated seroprevalence rates in the Americas due to natural infection to be between 0–3.2% in 2020 [7] with a drastic increase to 31.6–36% by March 2022 [8]. With the addition of wide spread vaccination, many current studies identified on SeroTracker, a global seroprevalence study dashboard, are unable to completely distinguish between natural infection and vaccination given the challenges with waning antibody [9]. In response, SARS-Cov-2 serology testing allows detection of the otherwise unapparent, yet equally-contagious cases not captured with testing using molecular diagnostic methods [3, 10, 11]. The role of serological testing therefore becomes important, in that determination of seroprevalence confers improved estimation of infection burden, enhances COVID risk analyses, and aids in the understanding of population exposure and immunity. The objective of the Manitoba COVID Seroprevalence (MCS) study was to evaluate the SARS-CoV-2 population seroprevalence of the Canadian province of Manitoba by conducting a population-based serosurvey over time.

## Methods

Study Population & design—Manitoba is a centrally located province in Canada. In 2020, the total population of Manitoba was 1,386,938, with 57% of the province’s population living in and around the Winnipeg region (*N* = 791,284). As part of universal healthcare coverage offered in all Canadian provinces, routine serological testing (ranging from prenatal screening to testing for specific pathogens – such as those for sexually transmitted and bloodborne infections) is provided free-of-charge to Manitoba citizens. The Manitoba MCS study is a population-based cross-sectional study initiated in April 2020 at Cadham Provincial Laboratory in Manitoba, Canada to measure the prevalence of SARS-CoV-2 antibodies across the province. Cadham Provincial Laboratory is the sole public health laboratory in Manitoba and routinely tests approximately 16,000 serum specimens per month from across the province. All residual specimens were eligible for inclusion in the study except specimens under investigation for SARS-CoV-2 related disorders such as multi-inflammatory syndrome in children and adults (MIS-C, MIS-A). Basic demographic information was collected from de-identified specimens including; sex, age, regional health authority, postal code, and vaccination status (including dose dates) for epidemiological analysis (Supplemental Fig. 1). This study was reviewed by the University of Manitoba Research Ethics board as routine surveillance and exempt under the Canadian Tri-Council Policy Statement: Ethical conduct for research involving humans, TCSP2.

Procedures & Laboratory Analysis – Approximately 1000 residual specimens were selected each month from April 1, 2020 – February 31, 2022 proportional by percentage for sex (50:50), age (20:20:20:20:20), and regional health authority (60:10:10:10:10). Manitoba is divided into 5 regional health authorities that represent different geographical areas of the province which include the Winnipeg Regional Health Authority, Northern Regional Health Authority, Southern Health-Sante Sud, Prairie Mountain Health, and Interlake-Eastern Regional Health Authority. 14,901 specimens were tested for SARS-CoV-2 IgG antibodies using clinically validated and Health Canada approved assays for antibodies directed against nucleocapsid (Abbott) and spike (DiaSorin) antigens. An algorithm was generated to identify antibodies resulting from natural infection or total antibody using vaccination history extracted from the provincial immunization registry (Supplemental Fig. 2). Natural infection was defined as a positive nucleocapsid, or spike antibody response in the absence of vaccination, whereas total antibody was estimated based on a positive antibody response from nucleocapsid or spike regardless of vaccination status. Both the nucleocapsid (sensitivity: 94.4%; specificity: 99.6%) and spike (sensitivity: 95.7%: specificity: 98.9%) IgG assays were previously validated by a third-party national evaluation [12]. A positive signal/noise cut-off of ≥ 0.7 and ≥ 15 were used for the Abbott nucleocapsid and DiaSorin spike IgG assays, respectively. Sampling months were determined using a parametric accelerated failure time model based on the daily PCR confirmed counts adjusted for symptom onset [3, 13]. This model makes use of seroconversion rates within the seroprevalence study to model the time from symptom onset to seroconversion and predicting when seroprevalence would begin to increase in the population after each wave.

### Statistical analysis

Using the R statistical computing package including; survey (v.3.4.0), dplyr (v1.1.2), tidyverse (v1.3.2) and ggplot (v3.4.0) packages, raw seroprevalence rates were calculated and weighted by sex, age, and regional health authority. Additionally, the results were corrected for the combined sensitivity and specificity of each antibody assay with 95% confidence intervals (95% CI) calculated using the exact binomial method. In order to correct for waning immunity in the population in cumulative estimate of prevalence, we used a previously published model-based approach that assumes the probability of sero-reversion for a given specimen decays exponentially over time [14]. This approach estimated the decay rate and proportion of the tested population that seroreverted, minimizing the number of new cases in October 2021 (a period with low case incidence), when changes in seroprevalence would principally have been due to waning antibody. This model also assumed that the probability of an individual seroreverting, decays exponentially over time. Parameters for this model were learned using the raw prevalence in each month assuming there were relatively few infections in October 2021 (10,000 iterations). Infection fatality ratios were calculated using the cumulative model-based prevalence estimates by sex, age, and regional health authority for each sampling month, with or without primary care home (PCH) deaths. Primary care homes are licensed residential facilities that provide services to individuals who can no longer manage independently at home or with family and often includes individuals of advanced age with chronic illness or disability. Deaths in Manitoba were defined as SARS-CoV-2 being the initial cause or contributing cause of death. Accounting for primary care home deaths allow us to improve our infection fatality estimates for the elderly that live in the community as appose to requiring care due to advanced age or illness.

## Results

**Fig. 1:**
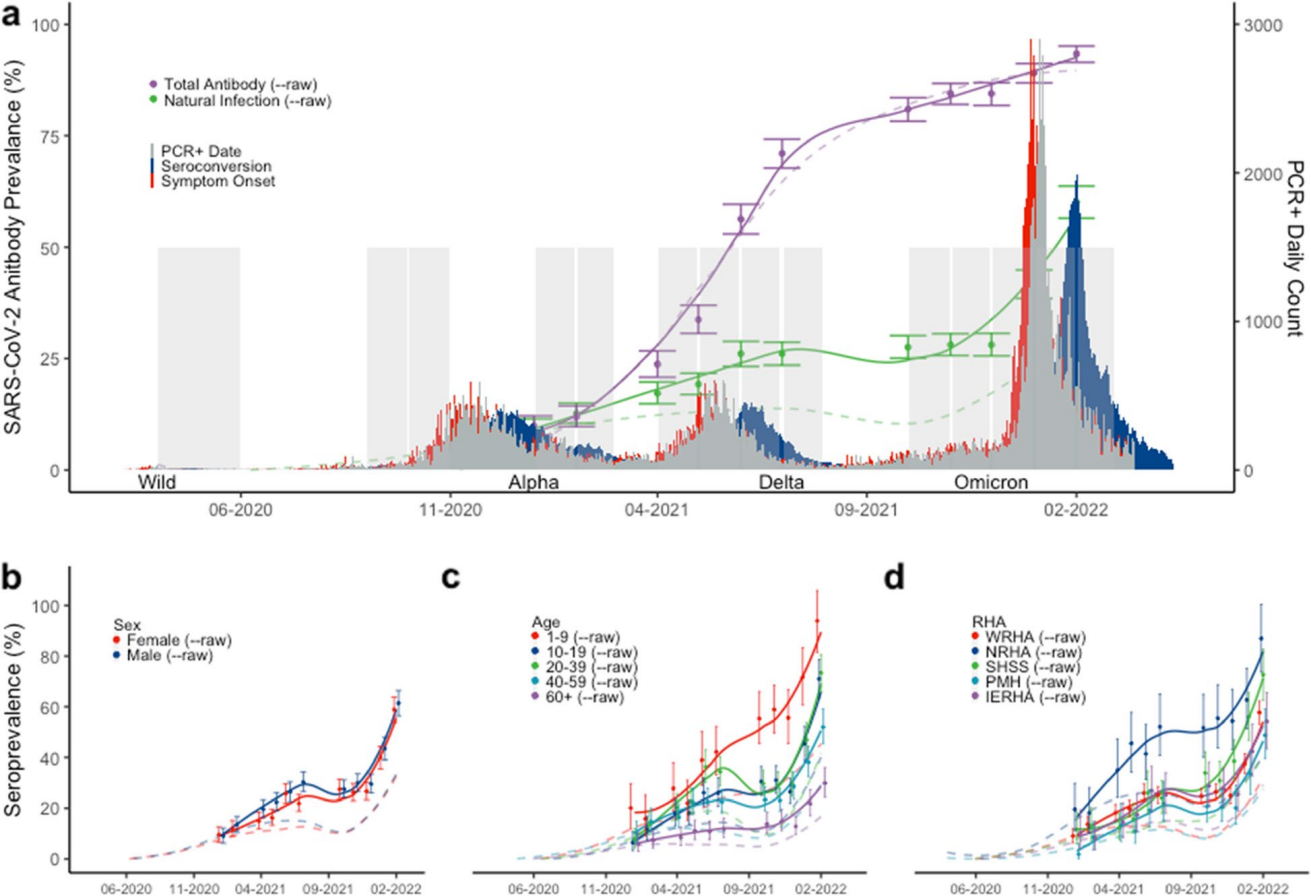
Seroprevalence in Manitoba, Canada during 4 pandemic waves. SARS-CoV-2 PCR-positive reporting dates (grey) were plotted and then backdated by 6 days to estimate symptom onset dates (red). Lab validation data was used to extrapolate and plot the expected seroconversion waves (blue). Sampling months outlined in grey boxes with total antibody (solid purple) and natural infection (solid green) seroprevalence rates for Manitoba are depicted along with raw rates (dashed). Raw and corrected seroprevalence rates are also depicted by sex (**b**), age (**c**), and regional health authority (**d**). Winnipeg Regional Health Authority (WRHA); Northern Regional Health Authority (NRHA); Southern Health-Sante Sud (SHSS); Prairie Mountain Health (PMH); Interlake-Eastern Regional Health Authority (IERHA); Error bars depict 95% confidence intervals

The MCS study was initiated in April 2020 shortly after serological assays were both approved and available for use. Initial raw prevalence in Manitoba was estimated to be 0–0.004% (95% CI) following the first nominal wave in April—May of 2020 (Fig. 1A). Continued sampling saw a significant increase in prevalence following the second wave from 1.10% (95%CI – 0.11–2.45) in October 2020 to 9.25% (95CI – 7.20–11.58) in January 2021. A third wave of the pandemic occurred shortly after, between April and July of 2021, however, raw prevalence increased by only 3.02% to 12.27% (95%CI – 10.46–15.23) by the end of July 2021, despite being a similar size to the second wave. Further analysis of nucleocapsid responses from repeat sampling within the MCS study indicates the half-life of these antibodies to be only 53 days (Supplemental Fig. 3) [5, 14, 15].

In order to account for waning immunity and provide a more accurate estimate of cumulative exposure and infection, we used a previously published exponential decay model [13, 14]. Using this approach, we were able to adjust our estimates to provide a cumulative seroprevalence estimate that more accurately predicted the proportion of infected individuals in the province following all four pandemic waves (Fig. 1A-raw vs. corrected). After correcting for waning immunity, the study showed a consistent rise in seroprevalence through the first three waves of the pandemic followed by a sharp rise in January – February 2022 to 60.14% (95%CI – 56.56–63.71). This equates to 6.3 SARS-CoV-2 infections for every PCR confirmed case following the omicron wave, or 3 in every 5 Manitobans having been infected.

The total antibody estimate for the province (measured by either nucleocapsid or spike antibody, irrespective of vaccination status) reached 93.4% (95%CI – 91.5–95.1) post-Omicron in February 2022. Waning immunity over time, with respect to nucleocapsid antibody, could be observed following each major wave (Supplemental Fig. 4A). In contrast, spike antibody levels on a population level mimicked the provincial vaccine roll-out peaking in July of 2021 (Supplemental Fig. 4B). Of interest, spike antibody responses on a population level started to decline leading up to the start of the Omicron wave in December of 2021, however, after this wave, levels have reached their highest point to date.

A more detailed analysis of sex, age, and regional health authority over time was conducted in order to identify risk factors to infection. Men and women had similar seroprevalence rates preceding the second wave of the pandemic, however following the third wave in July 2021, a disparity emerged with men having a higher prevalence [30.32% (95%CI: 26.50 – 34.34)] compared to women [21.89% (95%CI – 18.75–25.34)]. While the gap between men and women post-omicron has decreased, men continue to demonstrate 2.51% higher seroprevalence than women as of February 2022 (Fig. 1B). While seroprevalence increased overall, younger age groups had even higher prevalence. Children, 1–9 years old had the highest cumulative prevalence following an exponential trend throughout the pandemic, achieving 93.89% (95% CI – 81.35- 105.96) in February 2022 (Fig. 1C). Individuals aged 60 years and above consistently had the lowest prevalence during the pandemic reaching 29.90% (95%CI – 24.41–35.98). The 10–19 and 20–39 age groups had similar prevalence rates following the omicron wave of 70.97% (95%CI – 63.46–78.54) and 73.35% (95CI%—65.86–80.59), respectively. Interestingly, despite controlling for waning immunity, the 20–39 age category had a significant drop in prevalence just prior to the omicron wave (October 2021), whereas other age categories had a less pronounced or slight flattening of prevalence during the same time period.

The Northern Health Region in Manitoba had the highest cumulative seroprevalence rate throughout the pandemic, reaching 86.87% (95%CI – 73.58–100.39) in February 2022 (Fig. 1D). While relatively stable during the first two waves of the pandemic, the Southern Health Region began to show signs of community transmission in July 2021, leading to the second highest prevalence in the province at 72.53% (95%CI: 62.69–82.74) by February 2022. Mapping the raw seroprevalence incidence throughout the pandemic revealed pockets of transmission occurring in the Southern Health region in the summer of 2021 around the towns of Winkler, Morden, and Steinbach. The Winnipeg Health Region, which accounts for 70% of Manitoba’s population was similar to the provincial average, at 57.72% (95%CI – 53.03–62.33). In addition, seroprevalence was not evenly distributed within the city of Winnipeg, with significantly more prevalence occurring around the Downtown, and Point Douglas core areas of the city (Fig. 3 and Supplemental Video).

**Fig. 2:**
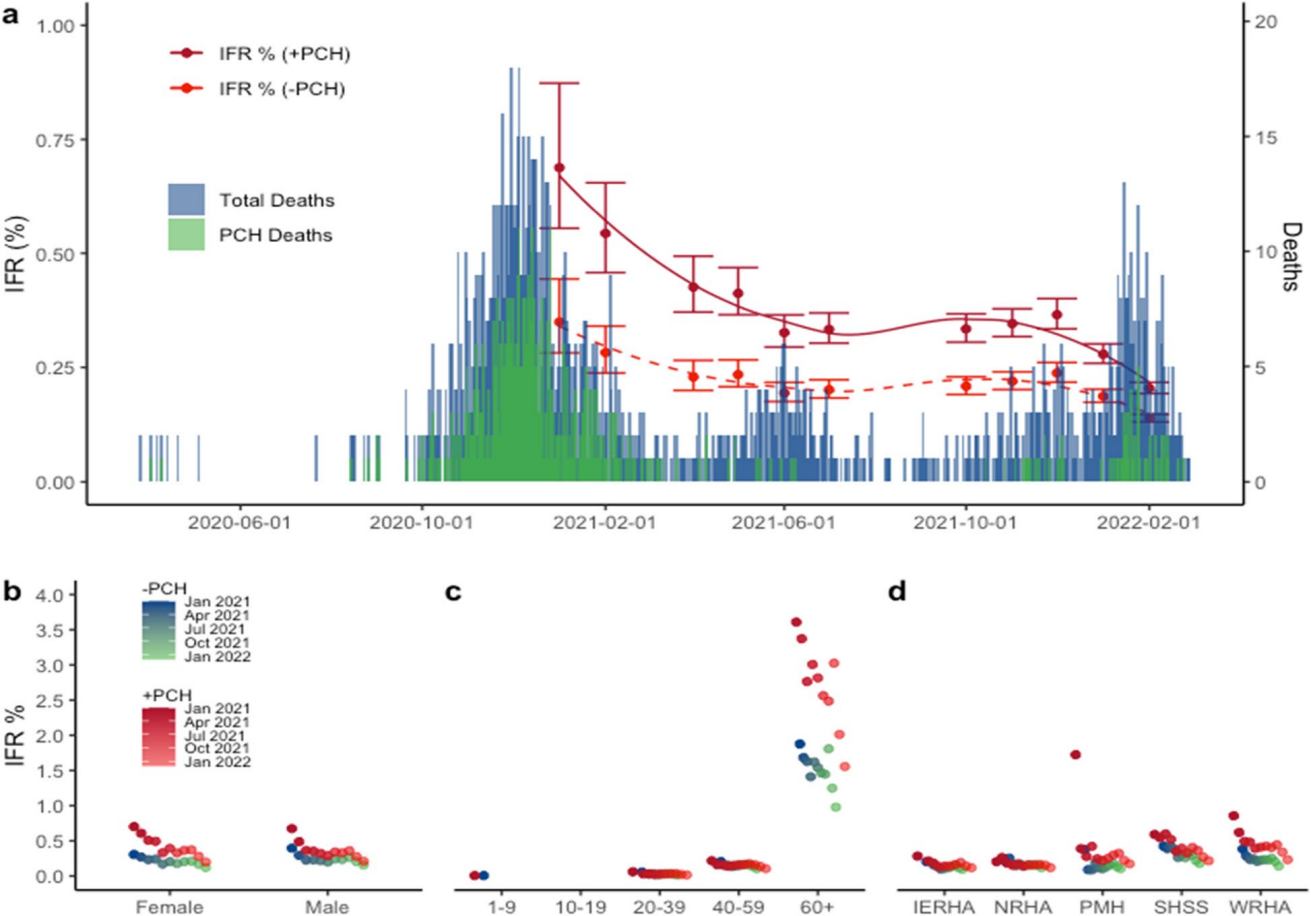
Infection fatality ratio estimates for Manitoba during 4 pandemic waves. Reported deaths (blue) and primary care home deaths (green) are illustrated along with the overall infection fatality ratio including (solid red) and excluding (dashed red) primary care home deaths. IFR estimates are also illustrated by sex (**b**), age group (**c**), and regional health authority (**d**). IFR rates for regional health authority are presented as crude rates. Winnipeg Regional Health Authority (WRHA); Northern Regional Health Authority (NRHA); Southern Health-Sante Sud (SHSS); Prairie Mountain Health (PMH); Interlake-Eastern Regional Health Authority (IERHA); Error bars depict 95% confidence intervals

Using the cumulative prevalence estimates, the overall infection fatality ratio for Manitoba (IFR) steadily declined from 0.69% (95%CI 0.55–0.87) in January 2020 to 0.20% (95%CI 0.19–0.22) following the Omicron wave in February 2022. When excluding primary care home deaths, the provincial IFR following Omicron was 0.14% (95%CI 0.13–0.15) (Fig. 2). IFR estimates increased exponentially with age (Fig. 2C). All age groups experienced a steady decline in IFR during the pandemic with the 60 + category peaking at 1.88% (95%CI 1.12–3.72) after the second wave and declining to 0.97% (95%CI 0.81–1.20) post-Omicron (excluding deaths in PCHs). Outside the Winnipeg Health Region, the Southern Health Region had the second highest IFR in the province 0.42% (95%CI 0.27–0.80) after the second wave and culminating in a low of 0.18% (95%CI 0.16–0.21) post-Omicron. Of considerable interest, is that despite the highest prevalence rates in the province, the Northern health region, had one of the lowest IFRs at 0.12% (95%CI 0.10–0.14).

## Discussion

The results of this study have provided an important description of the Manitoba pandemic after four waves, including Omicron. Initial estimates in May 2020 indicated that the seroprevalence to SARS-CoV-2 was very low (0–0.004%). Community wide transmission during this time was quite low, with the majority of cases associated with foreign travel as public health measures were at their most restrictive point. While there have been 131,068 PCR confirmed infections as of Feb 2022 in Manitoba, our data estimates that 833,966 Manitobans have been infected with SARS-CoV-2. This estimate equates to 3 in 5 Manitobans having developed antibodies due to natural infection. With the roll-out of mass vaccination in the province during 2021, we estimate that 93.4% of Manitobans have acquired antibodies to SARS-CoV-2 as of Feb 2022 (natural infection or vaccination). Given these high rates of immunity within the population, it is tempting to suggest that hospitalizations and severe cases that lead to ICU admissions may be blunted in the face of a possible fifth wave in the summer of 2022. However, the biological variation associated with age related immunity [16], clinical outcomes [17], and waning antibody [18], coupled with population level dynamics including geographical differences, make the prospects of a return to normal unclear. It is not surprising that PCR testing captured only a small portion of the evolving epidemic, compounded by variability in disease severity, testing practices, and public health policy.

A more detailed analysis of sex, age, and regional health authority over time was conducted in order to identify trends in risk of infection. Men and women had similar prevalence rates during the first and second wave of the pandemic, however a divergence occurred, with men achieving higher prevalence of 31.1% (95%CI—27.3–35.2) compared to 22.6% (95%CI – 19.4–26.1) during the third wave in 2021. Of note, a recent study from British Columbia did not identify any meaningful differences in sex over the same time period [19]. The gap between men and women post-omicron has since converged, although men continued to have slightly higher prevalence than women. Public health restrictions were at their greatest during the first two waves of the pandemic; as with many provinces, however, the summer of 2021 saw an easing of restrictions, particularly with respect to back-to-work initiatives. The prevalence disparity between men and women during this time may be due to men having increased contacts outside the home associated with employment. In parallel, extended closures following school holidays may have led women to disproportionately take on heightened childcare duties [20, 21]. In-person schooling and extracurricular activities were suspended in March 2020, and re-opened in September 2020 to significant health precautions including masks, cohorts, and reduced overall capacity in public buildings. Interestingly, during this time, women had a higher IFR than men despite the prevalence inversion (Fig. 2B), however after removing PCH deaths this disparity disappears. This may partially be explained by women having a disproportionate occupancy (67.6%) in Canadian care facilities [22].

We found that prevalence generally followed a decreasing trend with increasing age. Young children (1–9 years) had significantly higher prevalence than all other age groups, while the elderly (60 years and older) had the lowest. This trend has been observed globally as well as regionally in Canadian studies [7, 8, 19, 23]. Low prevalence in the elderly may be an indication of successful targeted public health measures to reduce transmission, however despite the relatively low prevalence, individuals 60 + years of age have the highest IFR (1.88%), highlighting other comorbidities that contribute to IFR. Alternatively, studies are also mounting that identify lower antibody levels in response to vaccination as well as natural infection in the elderly [16]. Including school age children is a major advantage in this study over previous studies and illustrates that despite what is seemingly high levels of infection, there were three lone deaths observed in Manitoba for this group throughout the pandemic. While many studies have observed lower nucleocapsid antibody responses, presumably due to mild clinical disease, our study showed children (1–9 years) had the highest median nucleocapsid response of all the age groups at a population level (Supplemental Fig. 5). One important limitation to consider in this study is that Cadham Provincial Laboratory proportionally receives limited samples from school aged children. It is conceivable that residual specimens from this age category may have a higher positive, pre-test probability, as they may be connected to the health care system for reasons other than routine screening. Additionally, estimating prevalence in infants 12 months or younger was problematic in that antibody positivity could conceivably be attributed to maternal natural immunity or vaccination, hence this age category was excluded from the provincial analysis [24]. Another potential limitation of our epidemiological seroprevalence study is the shifting use case given post-Omicron antibody prevalence is close to 100%. By capturing quantitative data, this study can continue to be relevant by detecting waning antibody in the community informing targeted vaccination strategies to prevent future outbreaks.

According to national data, Manitoba continues to have the second highest fatality rate in Canada due to COVID-19 with 121 deaths per 100,000 people, as of February 2022 [25]. However, when accounting for infections measured by seroprevalence, the Canadian infection-fatality ratio was estimated to be 0.67% in July 2021 [26], with provincial estimates of 0.80% and 0.59% for Ontario and British Columbia, respectively [27, 28]. Following Manitoba’s second wave at the beginning of 2021, the estimated infection fatality ratio was 0.69% (95%CI—0.55–0.87). It is clear that despite Manitoba’s high case fatality rate compared to other Canadian provinces, accounting for the natural infection prevalence reveals similar infection fatality rates as other Canadian provinces. We observed considerable geographical differences in prevalence throughout the province with the Northern Health Region achieving the highest prevalence due to natural infection 86.87% (95%CI – 73.58–100.39). Despite a non-essential travel restriction within the province of Manitoba beyond the 53^rd^ parallel, transmission in small / remote northern communities remained unchanged. However, despite high prevalence rates, the Northern Health Region had the lowest IFR in the province. Early prioritization of vaccination in remote Northern and Indigenous communities may have had a significant blunting effect on early IFR estimates in this scenario.

**Fig. 3:**
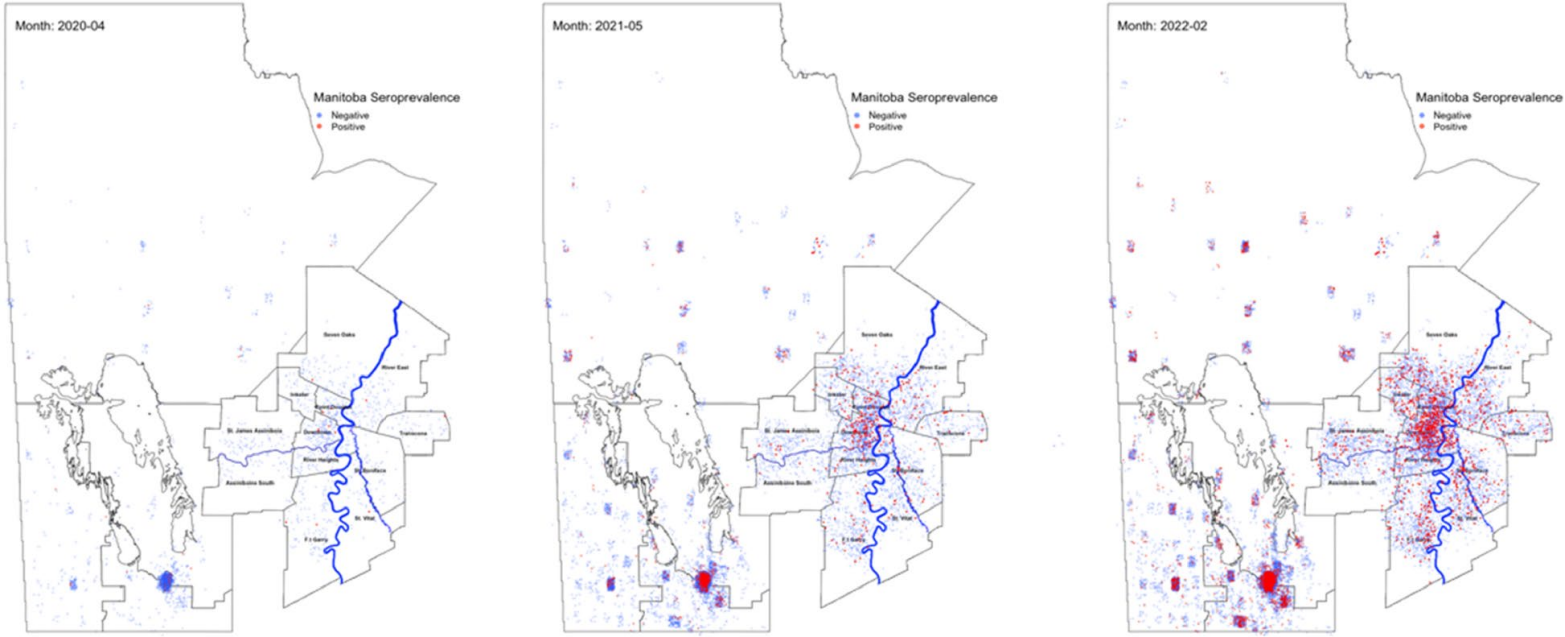
Geographical progression of seroprevalence in Manitoba, Canada and the greater Winnipeg area over 4 waves of the SARS-CoV-2 pandemic. Natural infections (red) and negative specimens (blue) are depicted for select months; April 2020 (**a**), May 2021 (**b**), and February 2022 (**c**)

The Southern health region had the second highest prevalence and highest reported IFR. Despite early success in limiting transmission in this region in 2020, significant pockets of transmission were observed in the summer of 2021 along with increased opposition to public health orders. Winnipeg, Manitoba’s most populous health region also experienced non-uniform prevalence in the lower income / housing dense areas of the city (Fig. 3 and Supplemental Video).

Our study has shown that despite high levels of immunity post-omicron, the humoral immune landscape to SARS-CoV-2 is constantly in flux. Continued monitoring of population trends over time will allow for informed data driven responses and policies, that allow for maximum public health impact with minimal intervention as we navigate a future post-COVID era.

### Supplementary Information

**Additional file 1: Supplemental Figure 1.** Demographic information including sex, age group and regional health authority of all participant specimens included in the study (total 14,901 specimens). **Supplemental Figure 2.** Algorithm that defines antibody response for natural infection as well as total immunity on a population level. **Supplemental Figure 3.** Nucleocaspid responses for specimens with repeat testing within the Manitoba COVID seroprevalence study. **Supplemental Figure 4.** S/CO (log10) of nucleocapsid and spike IgG antibody responses throughout the pandemic. **Supplemental Figure 5. **Nucleocapsid IgG antibody responses (log10) grouped by age category for specimens collected in the Manitoba COVID seroprevalence study.

**Additional file 2. **

## Data Availability

All summary data is public and available upon request to the Canadian COVID-19 Immunity Task Force. Analysis code is available upon reasonable request to the corresponding author.
