# Supplementary material for: Riding high: seroprevalence of SARS-CoV-2 after 4 pandemic waves in Manitoba, Canada, April 2020–February 2022
Source: BMC Public Health. 2023 Dec 5;23:2420. doi: 10.1186/s12889-023-17239-6 (PMC10696886; doi:10.1186/s12889-023-17239-6)
Supplement: Supplementary file 1 — Additional file 1: Supplemental Figure 1. Demographic information including sex, age group and regional health authority of all participant specimens included in the study (total 14,901 specimens). Supplemental Figure 2. Algorithm that defines antibody response for natural infection as well as total immunity on a population level. Supplemental Figure 3. Nucleocaspid responses for specimens with repeat testing within the Manitoba COVID seroprevalence study. Supplemental Figure 4. S/CO (log10) of nucleocapsid and spike IgG antibody responses throughout the pandemic. Supplemental Figure 5. Nucleocapsid IgG antibody responses (log10) grouped by age category for specimens collected in the Manitoba COVID seroprevalence study. [file 12889_2023_17239_MOESM1_ESM.docx]

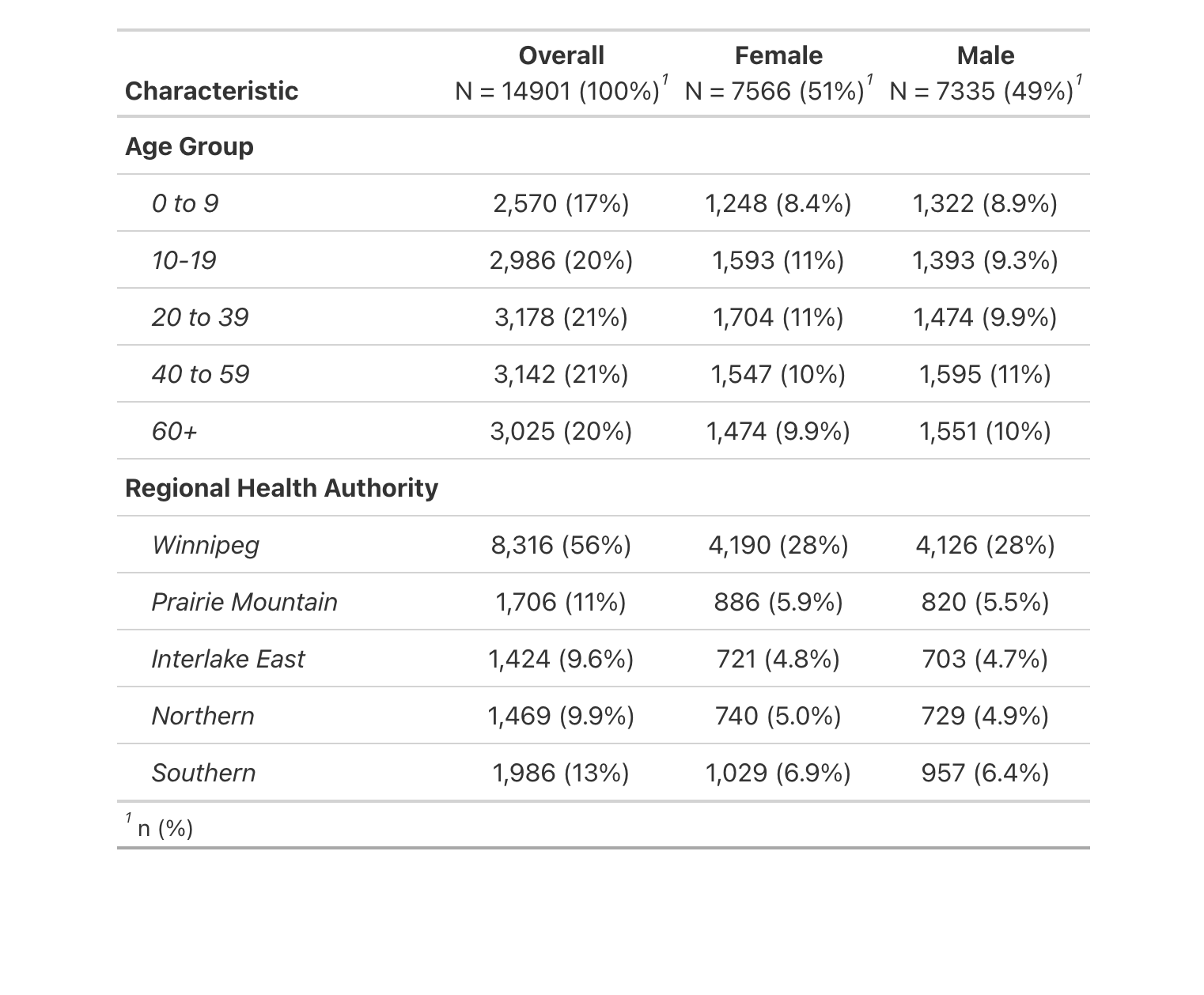


**Supplemental Figure 1.** Demographic information including sex, age group and regional health authority of all participant specimens included in the study (total 14,901 specimens).


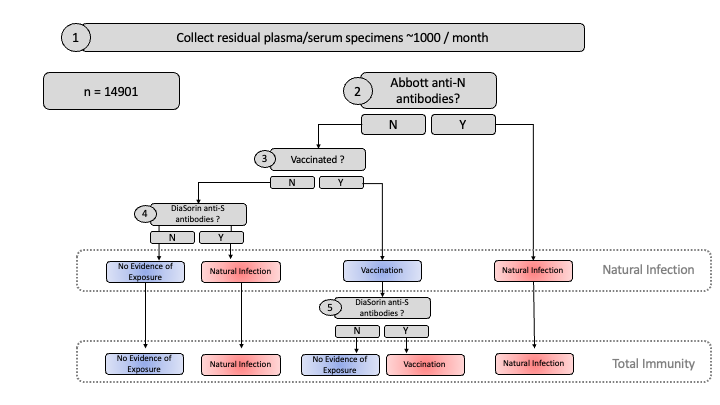


**Supplemental Figure 2.** Algorithm that defines antibody response for natural infection as well as total immunity on a population level.


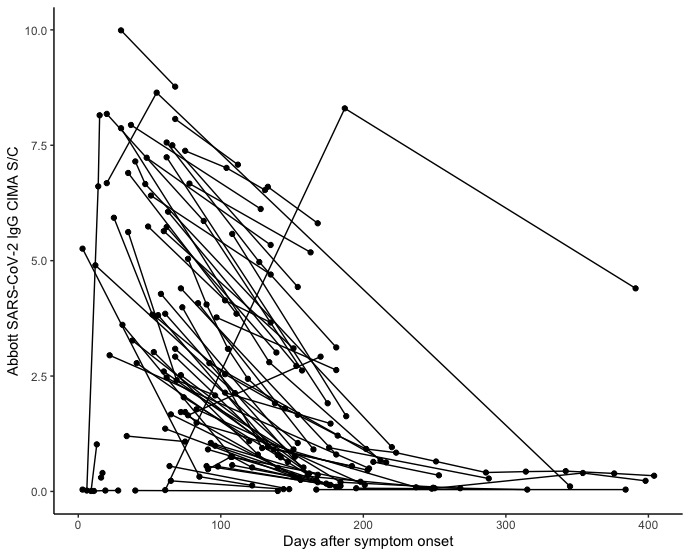


**Supplemental Figure 3.** Nucleocaspid responses for specimens with repeat testing within the Manitoba COVID seroprevalence study.


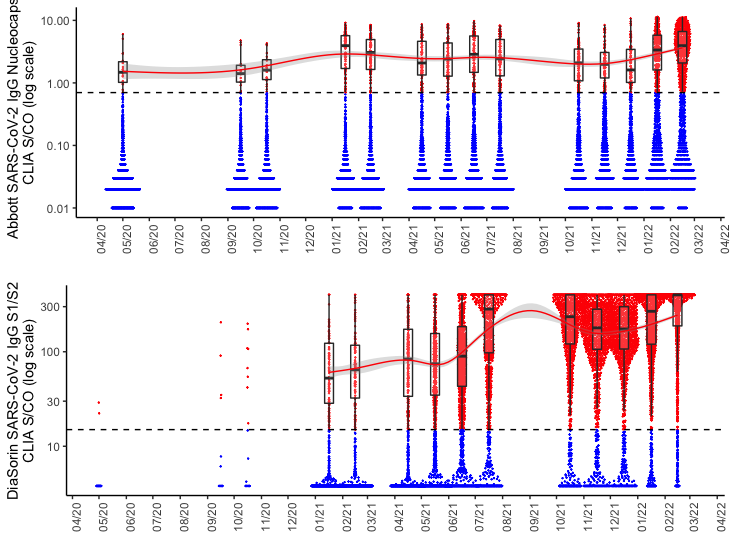


**Supplemental Figure 4.** S/CO (log10) of nucleocapsid and spike IgG antibody responses throughout the pandemic.

**f**

**
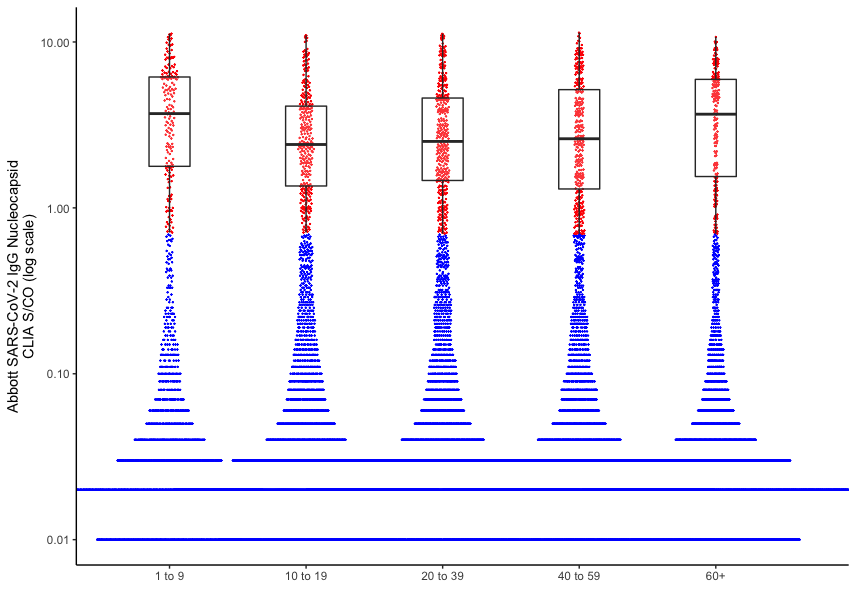
**

**Supplemental Figure 5.** Nucleocapsid IgG antibody responses (log10) grouped by age category for specimens collected in the Manitoba COVID seroprevalence study.
